# Evaluating the understandability and actionability of Japanese human papillomavirus vaccination educational materials on cervical cancer

**DOI:** 10.1093/heapro/daaf034

**Published:** 2025-04-23

**Authors:** Yuko Yamada, Tsuyoshi Okuhara, Rie Yokota, Emi Furukawa, Hiroko Okada, Takahiro Kiuchi

**Affiliations:** Department of Health Communication, Graduate School of Medicine, The University of Tokyo, Tokyo, Japan; Department of Health Communication, School of Public Health, Graduate School of Medicine, The University of Tokyo, Tokyo, Japan; Department of Medical Communication, School of Pharmacy and Pharmaceutical Science, Hoshi University, Tokyo, Japan; University Hospital Medical Information Network (UMIN) Center, The University of Tokyo Hospital, Tokyo, Japan; Department of Health Communication, School of Public Health, Graduate School of Medicine, The University of Tokyo, Tokyo, Japan; Department of Health Communication, School of Public Health, Graduate School of Medicine, The University of Tokyo, Tokyo, Japan

**Keywords:** health communication, health literacy, HPV vaccine, human papillomavirus, patient education, PEMAT

## Abstract

Educational materials about human papillomavirus (HPV) vaccination must be easy to understand and must support recommended behaviors regardless of readers’ health literacy levels. The purpose of this study was to evaluate the understandability, actionability, and comprehensiveness of HPV vaccination educational materials in Japan. From August to September 2023, we obtained HPV vaccination educational materials from the central government, local governments, and websites. We assessed the understandability and actionability of the materials using the Patient Education Materials Assessment Tool for Printed Materials (PEMAT-P), Japanese version. We also evaluated the comprehensiveness of the content. Ratings of understandability, actionability, and comprehensiveness were compared by material type and source. We evaluated 164 eligible materials. The mean understandability and actionability of all materials were 60.5% (standard deviation [SD] = 12.5) and 42.0% (SD = 20.5), respectively. Many materials lacked definitions of medical terms, clear explanations of numbers, content summaries, explicit steps of action, and the use of visual aids to improve understanding and actionability. The mean comprehensiveness score was 73.5% (SD = 14.7%). A few materials included all the necessary information content. The highest understandability score and actionability score were for local government mailings, and the highest comprehensiveness score was for academic materials. Most Japanese HPV vaccination educational materials were insufficiently understandable and actionable. Such materials need to be improved, especially regarding the use of numbers, medical terms, and visual aids. In terms of content, the importance of vaccination before sexual debut and the benefits of vaccination for men should be emphasized.

## Graphical Abstract

**Graphical Abstract:**
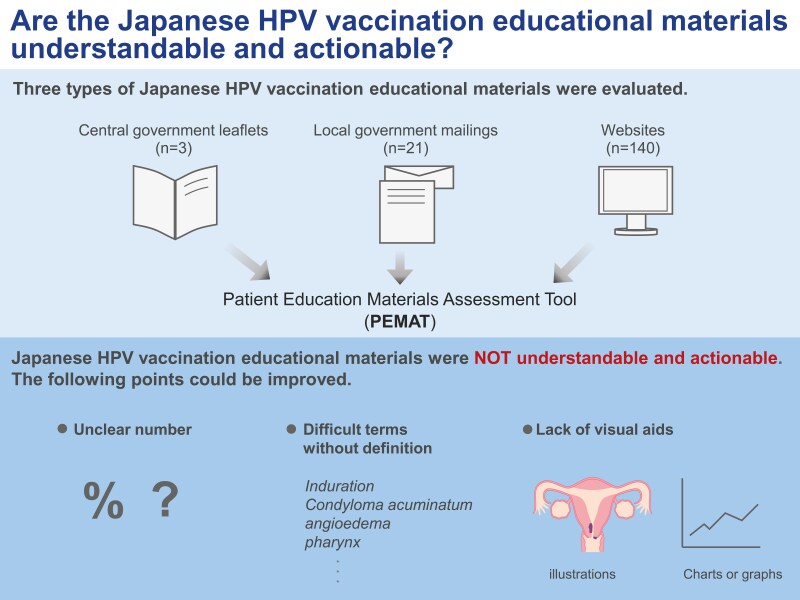

**Contribution to Health Promotion**

- Most Japanese HPV vaccination educational materials were insufficiently understandable and actionable and did not have all the necessary information content.
- Many materials need to be improved regarding the use of numbers, medical terms, and visual aids.
- The importance of vaccination before sexual debut and the benefits of vaccination for men should be included in the materials.

## BACKGROUND

Cervical cancer is caused by persistent high-risk human papillomavirus (HPV) infection and is the fourth most common cancer among women in the world (World Health Organization 2022). In Japan, there are approximately 11,000 new cases of cervical cancer and approximately 2900 deaths from the disease per year (National Cancer Center 2022). Fortunately, almost all cervical cancer is preventable. HPV vaccination is the primary preventive strategy and cancer screening for precancerous lesions is the secondary strategy (World Health Organization 2023). As part of its 2020 Global Strategy for the Elimination of Cervical Cancer, the World Health Organization has prompted countries to ensure that 90% of girls are fully vaccinated against HPV by the age of 15 years to meet the goal of an incidence rate of less than 4 per 100,000 women (the elimination standard) (World Health Organization 2020).

In Japan, HPV vaccination became a routine preventive measure for girls in grades 6–10 (from sixth grade of elementary school to first grade of high school) under the April 2013 Immunization Act (Ikeda et al. 2019, Ministry of Health, Labour and Welfare 2021a). However, active recommendations for routine HPV vaccination were subsequently discontinued for almost 9 years because of negative media reports of the adverse effects of HPV vaccination (Jwa et al. 2023). Following the demonstration of the efficacy and safety of the HPV vaccine by large-scale epidemiological studies in Japan (Suzuki and Hosono 2018, Matsumoto et al. 2019), active HPV vaccine recommendations were resumed in April 2022 (Ministry of Health Labour and Welfare 2021b). However, HPV vaccination coverage remains stagnant at 30.2% (fiscal year 2022, for the total population in the standard vaccination age range receiving three doses) (Ministry of Health, Labour and Welfare 2023a). Most routine childhood vaccinations, including measles, tuberculosis, and diphtheria, have a high coverage rate of more than 90% in Japan (Ministry of Health, Labour and Welfare 2023b). However, many parents do not have a positive attitude toward vaccination and lack an understanding of issues such as vaccination risks and benefits (Jwa et al. 2023). Parents exposed to negative information about vaccines may be reluctant to have their children vaccinated because of a lack of confidence in the vaccines (Jwa et al. 2023). It is therefore important that the relevant target population (and the parents of children in the target population) have a good understanding of HPV vaccination.

In its Healthy People 2030 initiative, the US Department of Health and Human Services updated the definition of health literacy and emphasized the importance of organizational as well as personal health literacy (Santana et al. 2021). In the new definition, personal health literacy is defined as ‘the degree to which individuals have the ability to find, understand, and use information and services to inform health-related decisions and actions for themselves and others’, and organizational health literacy is defined as ‘the degree to which organizations equitably enable individuals to find, understand, and use information and services to inform health-related decisions and actions for themselves and others’ (Santana et al. 2021). Thus, organizations such as public health institutions and local governments should be accountable for health literacy when creating and delivering health information. If health information providers deliver information that is difficult to understand or use, recipients will be unable to use it for optimal decision-making. Thus, health information providers should deliver health information that is easy to understand and actionable.

The understandability and actionability of health information have been evaluated using tools such as the Suitability Assessment of Materials (SAM) (Doak et al. 1996), the Patient Education Materials Assessment Tool (PEMAT) (Shoemaker et al. 2014), and readability assessment tools like the Flesch–Kincaid Reading Ease (FRE) test, Fry Readability Graph (FRG), Gunning Fog Index (GFI), Simple Measure of Gobbledygook Grade Level (SMOG), and Flesch–Kincaid Grade Level (FKGL) test (Friedman and Hoffman-Goetz 2006, Centers for Disease Control and Prevention 2009). One study evaluated the US Department of Health and Human Services HPV vaccine counseling materials using the SAM, PEMAT, and readability tools, and in terms of content comprehensiveness. The findings showed that most of the materials were too difficult to read, not understandable and actionable, and not suitable for the general public (Chhabra et al. 2018). Although the American Medical Association has recommended that patient materials are written at sixth-grade readability levels (American Medical Association Foundation and American Medical Association 2003), one scoping review of readability assessment studies reported that most HPV vaccine and cervical cancer materials were written at levels above sixth grade (Okuhara et al. 2021). Thus, previous research indicates the need to improve the understandability and actionability of HPV vaccination educational materials.

The PEMAT measures the understandability and actionability of health materials for patients and the general public. The PEMAT was developed by an expert team for the Agency for Healthcare Research and Quality in the USA. The validity and reliability of the PEMAT have been demonstrated (Shoemaker et al. 2014), and it is the most frequently used measure of its type. The PEMAT has been used to evaluate a wide range of patient education materials, such as those on genetic test results (Dwyer et al. 2021), COVID-19 home care (Furukawa et al. 2023), coronary artery bypass graft surgery (McLaughlin 2019), and HPV vaccine counseling materials (Chhabra et al. 2018). A Japanese version of the PEMAT has been developed and its reliability and validity have been confirmed (Furukawa et al. 2022). Although previous studies have assessed the readability of online Japanese HPV vaccination information, there are no comprehensive quantitative evaluations of the understandability and actionability of this information in Japan. This study aimed to assess the understandability, actionability, and content comprehensiveness of Japanese HPV vaccination educational materials, and to provide useful information to guide future improvements to such materials. The following research questions were investigated: (i) What are the levels of understandability and actionability of Japanese HPV vaccination educational materials? Which aspects of the materials are particularly difficult to understand and act upon? (ii) How comprehensive are the materials, and which types of content are not included? (iii) Are there any differences in PEMAT scores and comprehensiveness by material type and source?

## METHODS

### Material collection

We collected Japanese HPV vaccination educational materials between 15 August and 12 September 2023. In Japan, HPV vaccination is a routine preventive measure. Local governments take the initiative in sending information about HPV vaccination to two eligible subpopulations: one is the regular vaccination population of girls in grades 6–10 (sixth grade of elementary school to first grade of high school), and the other is a catch-up population of women who missed HPV vaccination during the period of June 2013 to March 2022, when active recommendations were suspended. In addition to their own materials, local governments often use HPV vaccination education leaflets created by the Japanese Ministry of Health, Labour and Welfare (MHLW) for local governments and local hospitals. Therefore, we collected both central government MHLW leaflets and mailings that local governments had sent to the eligible population. Online HPV vaccination materials were also examined because the Internet is one of the main sources of health information for Japanese people (Ministry of Health, Labour and Welfare 2014).

The following inclusion criteria for HPV vaccination educational materials were used. As mentioned in the Background, the target population for regular vaccination is girls in grades 6–10 and the HPV vaccination is referred to ‘cervical cancer preventive vaccination’ in Japan. Thus, HPV vaccination information materials provided by local governments are also intended to inform girls about cervical cancer. Taking this specific situation in Japan into account, we decided on the inclusion criteria: (i) the materials had to provide general HPV vaccination information (including an overview of cervical cancer and an overview of the HPV vaccine as a minimum), (ii) be written in the Japanese language, (iii) for the general public, (iv) related to regular vaccination, and (v) sent in the fiscal year 2023 (in case of local government mailings). We excluded materials that addressed only one aspect of HPV vaccination, such as only information about the 9-valent vaccine or side effects. We also excluded materials targeted only at the catch-up subpopulation; this was to avoid duplicate evaluations as most of the content on regular vaccination and catch-up vaccination overlaps.

#### Central government MHLW leaflets

We performed an online search using the terms ‘Ministry of Health, Labour and Welfare HPV leaflet’, and identified the MHLW leaflets on HPV vaccination on the MHLW webpage ‘Information materials on HPV vaccine’ from the search results (Ministry of Health, Labour and Welfare 2023c). Of the seven leaflets available on the MHLW website, six were extracted (the exception was a leaflet written for healthcare professionals).

#### Local government mailings

From August 15 to 18, 2023, we emailed and telephoned the 35 local governments with populations of more than half a million to request them to send us the printed HPV vaccination materials they had sent to the target population. These materials included such as HPV vaccination information documents, vaccine recommendation postcards, information about vaccination sites (e.g. hospital lists), and vaccine screening questionnaires.

#### Websites

We conducted an online search between 31 August and 12 September 2023, using Google Japan and Yahoo Japan. Approximately 75% and 14% of all online searches in Japan are conducted using these two sites, respectively (as of August 2023) (GlobalStats 2023).

First, we identified four main search terms: ‘HPV vaccine’, ‘HPV vaccination’, ‘cervical cancer vaccine’, and ‘cervical cancer vaccination’, with reference to a previous study (Okuhara et al. 2017). The term ‘human papilloma virus’ was not used because a comparison of the popularity trends of different terms using Google Trends showed that the latter term was used much less frequently than ‘HPV’ or ‘cervical cancer’.

Second, we selected several modifier terms with reference to previous studies, a Google Trends search, and combinations suggested by the searches (i.e. additional modifying terms associated with the four main online search terms). The terms ‘dangerous’, ‘risk’, ‘approval’, ‘disapproval’, ‘safety’, ‘side effect’, ‘receive’, and ‘not receive’ were identified from two previous studies (Fu et al. 2016, Okuhara et al. 2017). The term ‘side reaction’ was excluded at this point because Google Trends showed that it was less popular than ‘side effect’. Next, we checked the related keywords generated in Google Trends for each of the four above-mentioned main terms and extracted a new modifier, ‘male’, which the search showed had over 10% popularity. Google and Yahoo searches of the four main terms identified several suggestions for alternative terms and additional modifiers: ‘nine-valent’, ‘type’, ‘age’, ‘painful’, and ‘effect’. Finally, the 4 main keywords and 14 modifiers were selected as indicated in Table 1. We conducted 60 online searches (4 for the main terms and 56 for combinations of the 4 main terms and the 14 modifiers) using Google and Yahoo.

**Table 1.** Website search terms

No.	Main search terms	Modifiers
1	HPV vaccine	male
2	HPV vaccination	side effect
3	cervical cancer vaccine	nine-valent
4	cervical cancer vaccination	type
5		age
6		painful
7		dangerous
8		safety
9		effect
10		risk
11		approval
12		disapproval
13		receive
14		not receive

We entered each identified query into the search windows one by one without our institutional network and the personal account of Google or Yahoo to minimize search bias. The top 50 websites of search results were extracted and those URLs were recorded in Microsoft Excel. We then excluded websites with the following characteristics based on the above-mentioned inclusion criteria: duplicate sites, news and newspaper articles, blogs/announcements/clinic news containing personal opinions, video sites, advertisements, Q&A-only sites, sites for medical professionals, sites that could not be accessed (e.g. link not found, paid access, member registration required for access), webpages of images, Yahoo answers, and sponsor sites.

### Coding procedures

We assessed the basic characteristics, understandability, actionability, and comprehensiveness of the collected HPV vaccination educational materials and recorded scores using a coding sheet created in Excel. The coding procedure was as follows.

#### Coding unit

For the MHLW leaflets, each leaflet was considered to be one coding unit. For the local government mailings, all materials contained in the mailing were considered to be one unit; for example, if the local government sent an HPV vaccine information document, a hospital list, and a MHLW leaflet in one mailing, these three materials were evaluated as one unit. For websites, one unit includes all pages about HPV vaccination on one website.

To obtain page counts of the printed materials, an A4-size document page was counted as one page. For websites, a webpage counts as one page.

#### Evaluation of understandability and actionability

We scored the understandability and actionability of materials using the Japanese version of the PEMAT for printable materials (PEMAT-P), which has demonstrated validity and reliability (Furukawa et al. 2022). The Japanese version of the PEMAT-P assesses health materials for patients and the general public according to whether each of the 16 understandability criteria and 7 actionability criteria are satisfied (Furukawa et al. 2022). Items are scored as agree = 1, disagree = 0, not applicable = N/A; the total score is calculated as a percentage by dividing the number of total ‘agree’ responses by the total possible items, excluding non-applicable items. We used a cutoff threshold of 70% to determine the understandability and actionability of the material, as set by the Agency for Healthcare Research and Quality, which developed the original PEMAT (Shoemaker et al. 2014), and following previous studies. We also calculated the mean score on each item as a range from 0 to 1. Because smartphones are the most commonly used devices to connect to the Internet (Ministry of Internal Affairs and Communications 2023), we evaluated understandability and actionability after switching the computer screen to the smartphone view using the developer function of Google Chrome.

#### Evaluation of comprehensiveness

To determine the comprehensiveness of the HPV vaccination education materials, we first extracted contents as follows with reference to a previous study (Chhabra et al. 2018): (1) overview of cervical cancer, (2) HPV vaccine as cancer prevention, (3) emphasis on target grade for vaccination, (4) importance of vaccination before first sexual intercourse, (5) information about safety and efficacy, (6) explanation of the need for three doses of the vaccine, (7) no charge for vaccinations, (8) benefits of vaccination for men. We added (9) side effects or adverse reactions given the unique Japanese situation. Thus, nine types of content were identified. In the case of the MHLW leaflets and the local government mailings, content type (8) was evaluated as ‘not applicable’ because they were not intended to be read by men. We scored the comprehensiveness of content with reference to a previous study as follows: present = 1, absent = 0, not applicable = N/A. A percentage score was subsequently obtained by dividing the total number of ‘present’ items by the total number of possible items (excluding non-applicable items).

### Statistical analysis

Descriptive statistics was used to summarize the characteristics of the materials, understandability and actionability scores, and content comprehensiveness. We used the Kruskal–Wallis rank sum test to compare the scores on understandability, actionability, and comprehensiveness by material type (MHLW leaflet, local government mailing, and website) and material source (government, hospital, and academia); government materials took all three material types, and all the materials from hospital and academic sources were web-based. The ‘other’ group for material source was excluded from the comparisons because it comprised a mixture of different sources, such as profit-making companies, nonprofit organizations, and semigovernmental corporations. For comparisons that showed significant differences in the Kruskal–Wallis test, we conducted the Wilcoxon rank sum test for all group combinations and corrected the *P*-values using the Benjamini–Hochberg method. To assess understandability, actionability, and comprehensiveness, two raters (a nurse and a health communication researcher) individually assessed 20% of the materials after 2 h of coding training, and Gwet’s AC1 scores were calculated to assess interrater reliability. R (version 4.3.2) was used with a significance criterion of *P* = 0.05 (two-sided) for all statistical analyses.

## RESULTS

### Overview of the materials

**Figure 1.**
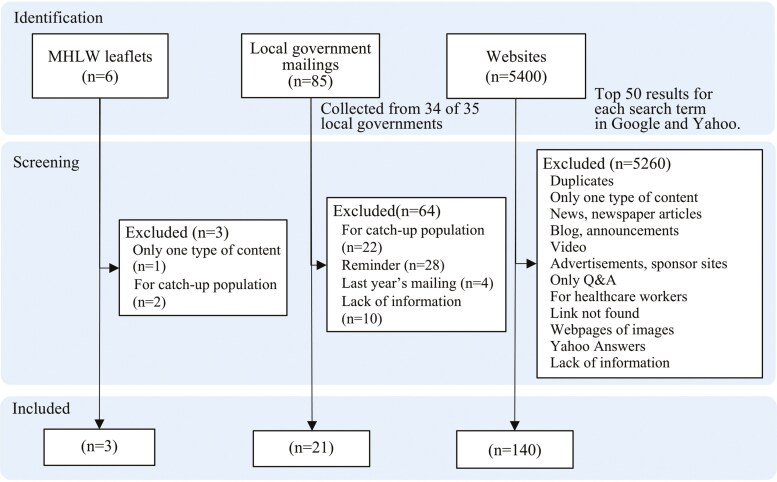
Selection of the materials.

Of the collected materials, 164 were eligible for assessment: 3 MHLW leaflets, 21 local government mailings, and 140 websites (Fig. 1). The characteristics of the materials are shown in Table 2. Websites accounted for 85.4% of the material types. Most of the materials came from the government (73.2%), followed by hospitals (17.1%), which together accounted for approximately 90% of all materials. Regarding the number of pages, most materials (66.5%) comprised just one page (minimum: one page; maximum: nine pages). The MHLW leaflets were used in 76.2% of the local government mailings and 52.1% of websites.

**Table 2.** Characteristics of materials

		MHLW		Local government		Web site		Total	
		(*n* = 3)		(*n* = 21)		(*n* = 140)		(*n* = 164)	
		*n*	%	*n*	%	*n*	%	*n*	%
Material shape									
	A4 document	3	100.0	21	100.0	0	0.0	24	14.6
	Web site	0	0.0	0	0.0	140	100.0	140	85.4
Material source									
	Government	3	100.0	21	100.0	96	68.6	120	73.2
	Hospital	0	0.0	0	0.0	28	20.0	28	17.1
	Academia	0	0.0	0	0.0	8	5.7	8	4.9
	Other	0	0.0	0	0.0	8	5.7	8	4.9
Topic									
	Target population	2	66.7	21	100.0	128	91.4	151	92.1
	Vaccination procedure	0	0.0	7	33.3	31	22.1	38	23.2
	Overview of cervical cancer	3	100.0	21	100.0	140	100.0	164	100.0
	Overview of HPV vaccine	3	100.0	21	100.0	140	100.0	164	100.0
	Vaccine type and alternate vaccinations	3	100.0	21	100.0	125	89.3	149	90.9
	Vaccination schedule	3	100.0	21	100.0	113	80.7	137	83.5
	Precautions for vaccination	0	0.0	18	85.7	44	31.4	62	37.8
	Resumption of positive recommendation	0	0.0	10	47.6	90	64.3	100	61.0
	Precaution after vaccination	3	100.0	21	100.0	111	79.3	135	82.3
	Consultation and contact information	2	66.7	20	95.2	107	76.4	129	78.7
	Accompanied by a parent	0	0.0	19	90.5	43	30.7	62	37.8
	Relief system for Vaccination	2	66.7	21	100.0	67	47.9	90	54.9
	Necessity of cervical cancer screening	3	100.0	20	95.2	113	80.7	136	82.9
	Vaccination interval with other vaccines	0	0.0	12	57.1	27	19.3	39	23.8
	No charge for vaccinations	0	0.0	17	81.0	82	58.6	99	60.4
	Out-of-district vaccination	0	0.0	11	52.4	47	33.6	58	35.4
Pages									
	1	0	0.0	0	0.0	109	77.9	109	66.5
	2	1	33.3	8	38.1	22	15.7	31	18.9
	3	0	0.0	2	9.5	5	3.6	7	4.3
	4	1	33.3	6	28.6	1	0.7	8	4.9
	5	0	0.0	2	9.5	0	0.0	2	1.2
	6	0	0.0	2	9.5	1	0.7	3	1.8
	7	0	0.0	1	4.8	1	0.7	2	1.2
	8	1	33.3	0	0.0	0	0.0	1	0.6
	9	0	0.0	0	0.0	1	0.7	1	0.6
Use of MHLW leaflet ^a^		-	-	16	76.2	73	52.1	89	54.3

^a^If a local government mailing included some MHLW leaflets in the mailing package or a website had a hyperlink to MHLW leaflets, this was counted as one use of MHLW leaflets. HPV, human papillomavirus; MHLW, Ministry of Health, Labour and Welfare.

### Understandability and actionability

The mean Gwet’s AC1 interrater reliability scores for understandability and actionability were 0.81 and 0.85, respectively (Supplementary File S1). The understandability and actionability scores on the Japanese version of the PEMAT-P are shown in Table 3 (by material type) and Table 4 (by material source). The mean understandability of all materials was 60.46% (standard deviation [SD] = 12.48), and the mean actionability was 41.97% (SD = 20.45). Of materials, 29 (17.7%) were evaluated as understandable and 13 (7.9%) were evaluated as actionable using the cutoff of 70%.

**Table 3.** Understandability, actionability, and comprehensiveness scores for HPV vaccination educational materials by material type

			MHLW		Local government		Web site		Total	
			(*n* = 3)		(*n* = 21)		(*n* = 140)		(*n* = 164)	
Item			Mean	SD	Mean	SD	Mean	SD	Mean	SD
**Understandability**			**59.72**	**6.01**	**66.09**	**8.03**	**59.63**	**12.93**	**60.46**	**12.48**
Content										
	1	The material makes its purpose completely evident	0.00	0.00	0.90	0.30	0.58	0.50	0.61	0.49
	2	The material does not include information or content that distracts from its purpose	0.67	0.58	0.81	0.40	0.86	0.35	0.85	0.36
Word choice and style										
	3	The material uses common, everyday language	1.00	0.00	0.71	0.46	0.46	0.50	0.51	0.50
	4	Medical terms are used only to familiarize audience with the terms. When used, medical terms are defined	0.33	0.58	0.24	0.44	0.29	0.45	0.28	0.45
Use of numbers										
	5	Numbers appearing in the material are clear and easy to understand	0.00	0.00	0.11	0.32	0.16	0.37	0.15	0.36
	6	The material does not expect the user to perform calculations	1.00	0.00	1.00	0.00	1.00	0.00	1.00	0.00
Organization										
	7	The material breaks or ‘chunks’ information into short sections	1.00	0.00	1.00	0.00	0.99	0.12	0.99	0.11
	8	The material’s sections have informative headers	1.00	0.00	1.00	0.00	0.94	0.25	0.95	0.23
	9	The material presents information in a logical sequence	1.00	0.00	1.00	0.00	0.94	0.23	0.95	0.22
	10	The material provides a summary	0.00	0.00	0.00	0.00	0.05	0.22	0.04	0.20
Layout and design										
	11	The material uses visual cues (e.g. arrows, boxes, bullets, bold, larger font, highlighting) to draw attention to key points	1.00	0.00	0.90	0.30	0.70	0.46	0.73	0.44
Use of visual aids										
	14	The material uses visual aids whenever they could make content more easily understood (e.g. illustration of healthy portion size)	0.00	0.00	0.05	0.22	0.07	0.26	0.07	0.25
	15	The material’s visual aids reinforce rather than distract from the content	0.00	0.00	0.58	0.51	0.83	0.38	0.77	0.42
	16	The material’s visual aids have clear titles or captions	0.67	0.58	0.95	0.23	0.71	0.45	0.75	0.44
	17	The material uses illustrations and photographs that are clear and uncluttered	1.00	0.00	0.84	0.37	0.43	0.50	0.51	0.50
	18	The material uses simple tables with short and clear row and column headings	1.00	0.00	0.38	0.50	0.52	0.50	0.51	0.50
**Actionability**			**38.89**	**9.62**	**54.92**	**18.31**	**40.10**	**20.29**	**41.97**	**20.45**
	19	The material clearly identifies at least one action the user can take	1.00	0.00	1.00	0.00	0.96	0.19	0.97	0.17
	20	The material addresses the user directly when describing actions	0.00	0.00	0.67	0.48	0.31	0.46	0.35	0.48
	21	The material breaks down any action into manageable, explicit steps	0.00	0.00	0.24	0.44	0.19	0.39	0.19	0.39
	22	The material provides a tangible tool (e.g. menu planners, checklists) whenever it could help the user take action	0.00	0.00	0.52	0.51	0.32	0.47	0.34	0.48
	23	The material provides simple instructions or examples of how to perform calculations	NA	NA	NA	NA	NA	NA	NA	NA
	24	The material explains how to use the charts, graphs, tables, or diagrams to take actions	1.00	0.00	0.94	0.24	0.89	0.32	0.90	0.30
	25	The material uses visual aids whenever they could make it easier to act on the instructions	0.33	0.58	0.00	0.00	0.04	0.20	0.04	0.20
**Content comprehensiveness**			**83.33**	**7.22**	**86.31**	**8.75**	**71.35**	**14.48**	**73.48**	**14.69**
	1	Overview of cervical cancer	1.00	0.00	1.00	0.00	1.00	0.00	1.00	0.00
	2	HPV vaccine as cancer prevention	1.00	0.00	1.00	0.00	0.97	0.17	0.98	0.15
	3	Emphasis on vaccination for girls at grades 6–10	1.00	0.00	1.00	0.00	0.91	0.29	0.92	0.27
	4	Importance of vaccination before first sexual intercourse	0.00	0.00	0.19	0.40	0.26	0.44	0.24	0.43
	5	Information about safety and efficacy	1.00	0.00	0.95	0.22	0.89	0.32	0.90	0.31
	6	Explanation of need for three vaccination doses	1.00	0.00	1.00	0.00	0.89	0.32	0.90	0.30
	7	No charge for vaccinations	0.67	0.58	0.76	0.44	0.61	0.49	0.63	0.48
	8	Benefits of vaccination for men	NA	NA	NA	NA	0.18	0.38	0.18	0.38
	9	Side effects or adverse reactions	1.00	0.00	1.00	0.00	0.73	0.45	0.77	0.42

HPV, human papillomavirus; PEMAT-P, Patient Education Materials Assessment Tool for Printed Materials; SD, standard deviation.

**Table 4.** Understandability, actionability, and comprehensiveness scores for HPV vaccination educational materials by material source

			Government		Hospital		Academia		Other		Total	
			(*n* = 120)		(*n* = 28)		(*n* = 8)		(*n* = 8)		(*n* = 164)	
Item			Mean	SD	Mean	SD	Mean	SD	Mean	SD	Mean	SD
**Understandability**			**63.19**	**9.63**	**52.24**	**14.65**	**44.38**	**18.08**	**64.38**	**13.42**	**60.46**	**12.48**
Content												
	1	The material makes its purpose completely evident	0.68	0.47	0.32	0.48	0.38	0.52	0.75	0.46	0.61	0.49
	2	The material does not include information or content that distracts from its purpose	0.89	0.31	0.71	0.46	0.63	0.52	0.88	0.35	0.85	0.36
Word choice and style												
	3	The material uses common, everyday language	0.58	0.50	0.25	0.44	0.13	0.35	0.63	0.52	0.51	0.50
	4	Medical terms are used only to familiarize audience with the terms. When used, medical terms are defined	0.29	0.46	0.14	0.36	0.13	0.35	0.75	0.46	0.28	0.45
Use of numbers												
	5	Numbers appearing in the material are clear and easy to understand	0.15	0.36	0.11	0.32	0.25	0.46	0.25	0.46	0.15	0.36
	6	The material does not expect the user to perform calculations	1.00	0.00	1.00	0.00	1.00	0.00	1.00	0.00	1.00	0.00
Organization												
	7	The material breaks or ‘chunks’ information into short sections	0.99	0.09	0.96	0.19	1.00	0.00	1.00	0.00	0.99	0.11
	8	The material’s sections have informative headers	0.98	0.13	0.86	0.36	0.63	0.52	1.00	0.00	0.95	0.23
	9	The material presents information in a logical sequence	0.98	0.16	0.82	0.39	1.00	0.00	1.00	0.00	0.95	0.22
	10	The material provides a summary	0.03	0.16	0.07	0.26	0.13	0.35	0.13	0.35	0.04	0.20
Layout and design												
	11	The material uses visual cues (e.g. arrows, boxes, bullets, bold, larger font, highlighting) to draw attention to key points	0.79	0.41	0.64	0.49	0.25	0.46	0.63	0.52	0.73	0.44
Use of visual aids												
	14	The material uses visual aids whenever they could make content more easily understood (e.g. illustration of healthy portion size)	0.08	0.26	0.04	0.19	0.13	0.35	0.00	0.00	0.07	0.25
	15	The material’s visual aids reinforce rather than distract from the content	0.79	0.41	0.73	0.46	0.57	0.53	0.83	0.41	0.77	0.42
	16	The material’s visual aids have clear titles or captions	0.84	0.37	0.59	0.50	0.29	0.49	0.50	0.55	0.75	0.44
	17	The material uses illustrations and photographs that are clear and uncluttered	0.54	0.50	0.55	0.51	0.14	0.38	0.33	0.52	0.51	0.50
	18	The material uses simple tables with short and clear row and column headings	0.52	0.50	0.62	0.51	0.33	0.52	0.00	0.00	0.51	0.50
**Actionability**			**45.92**	**19.14**	**29.88**	**20.38**	**37.50**	**14.56**	**29.58**	**25.35**	**41.97**	**20.45**
	19	The material clearly identifies at least one action the user can take	1.00	0.00	0.93	0.26	1.00	0.00	0.63	0.52	0.97	0.17
	20	The material addresses the user directly when describing actions	0.42	0.50	0.18	0.39	0.13	0.35	0.13	0.35	0.35	0.48
	21	The material breaks down any action into manageable, explicit steps	0.22	0.41	0.11	0.31	0.13	0.35	0.13	0.35	0.19	0.39
	22	The material provides a tangible tool (e.g. menu planners, checklists) whenever it could help the user take action	0.41	0.49	0.14	0.36	0.13	0.35	0.25	0.46	0.34	0.48
	23	The material provides simple instructions or examples of how to perform calculations	NA	NA	NA	NA	NA	NA	NA	NA	NA	NA
	24	The material explains how to use the charts, graphs, tables, or diagrams to take actions	0.93	0.26	0.71	0.49	1.00	0.00	0.75	0.50	0.90	0.30
	25	he material uses visual aids whenever they could make it easier to act on the instructions	0.03	0.16	0.07	0.26	0.13	0.35	0.13	0.35	0.04	0.20
**Content comprehensiveness**			**72.65**	**13.35**	**70.63**	**17.69**	**88.89**	**8.40**	**80.56**	**18.54**	**73.48**	**14.69**
	1	Overview of cervical cancer	1.00	0.00	1.00	0.00	1.00	0.00	1.00	0.00	1.00	0.00
	2	HPV vaccine as cancer prevention	0.98	0.16	0.96	0.19	1.00	0.00	1.00	0.00	0.98	0.15
	3	Emphasis of grade of 6–10 recommendation	0.96	0.20	0.71	0.46	1.00	0.00	1.00	0.00	0.92	0.27
	4	Importance of vaccination before first sexual intercourse	0.16	0.37	0.36	0.49	0.75	0.46	0.63	0.52	0.24	0.43
	5	Information of safety and efficacy	0.88	0.32	0.93	0.26	1.00	0.00	0.88	0.35	0.90	0.31
	6	Explanation of three times vaccination	0.92	0.28	0.89	0.31	0.88	0.35	0.75	0.46	0.90	0.30
	7	No charge for vaccinations	0.63	0.48	0.50	0.51	0.88	0.35	0.75	0.46	0.63	0.48
	8	Vaccination and benefit for men	0.07	0.26	0.32	0.48	0.50	0.53	0.63	0.52	0.18	0.38
	9	Side effects or adverse reactions	0.78	0.41	0.68	0.48	1.00	0.00	0.63	0.52	0.77	0.42

HPV, human papillomavirus; PEMAT-P, Patient Education Materials Assessment Tool for Printed Materials; SD, standard deviation.

A more detailed evaluation of understandability showed that none of the materials expected the reader to perform calculations (*n* = 164, 100%). Three items on the topic of organization were scored ≥ 0.95 (‘chunking’ information, use of informative headers, and presentation of information in a logical sequence). However, most materials did not define medical terms (*n* = 118, 72.0%), did not clarify the meaning of numbers (*n* = 140, 85.4%), did not contain a summary (*n* = 157, 95.7%), and did not use visual aids to make the content more understandable (*n* = 153, 93.3%). The evaluation of actionability showed that many materials indicated at least one action the user could take (*n* = 159, 97.0%), and explained simple instructions on how to use the charts, graphs, tables, or diagrams to take actions (if appropriate) (*n* = 76, 90.5%). However, most materials did not use visual aids to help the user act on instructions (*n* = 157, 95.7%) and did not explain the actions to be taken in terms of specific steps (*n* = 133, 81.1%).

A comparison of understandability and actionability by material type and material source is shown in Table 5. The comparison of material types showed no significant differences in understandability. However, the multiple comparisona showed that the actionability score for local government mailings was significantly higher than that for websites (median: 60.0, 40.0, respectively, adjusted *P* = 0.005). The comparison of material sources showed that the understandability score for government materials was significantly higher than that for academic materials (median: 62.5, 40.6, respectively, adjusted *P* = 0.001), and the score for government materials was higher than that for hospital materials (median: 62.5, 50.0, respectively, adjusted *P* < 0.001). The actionability score for government materials was higher than that for hospital materials (median: 40.0, 20.0, respectively, adjusted *P* < 0.001).

**Table 5.** Comparison of understandability, actionability, and comprehensiveness by material type and material source

				Understandability				Actionability				Comprehensiveness			
		*n*	%	Mean	SD	Median	*P*-value	Mean	SD	Median	*P*-value	Mean	SD	Median	*P*-value
Material type															
	MHLW	3	1.8	59.72	6.01	56.25	0.109	38.89	9.62	33.33	0.011^*^	83.33	7.22	87.50	0.000^***^
	Local government	21	12.8	66.09	8.03	66.67		54.92	18.31	60.00		86.31	8.75	87.50	
	Website	140	85.4	59.63	12.93	62.50		40.10	20.29	40.00		71.35	14.48	66.67	
Material source															
	Government	120	73.2	63.19	9.63	62.50	0.010^*^	45.92	19.14	40.00	0.000^***^	72.65	13.35	75.00	0.002^**^
	Hospital	28	17.1	52.24	14.65	50.00		29.88	20.38	20.00		70.63	17.69	66.67	
	Academia	8	4.9	44.38	18.08	40.63		37.50	14.56	33.33		88.89	8.40	88.89	

The Kruskal–Wallis test was used for comparisons by material type and issuance source. If there was a significant difference, a Wilcoxon rank sum test was performed for each combination, and *P*-values were corrected using the Benjamin–Hatchberg method. The *P*-values in the table are the results of the Kruskal–Wallis test. *P < 0.05, **P < 0.01, ***P < 0.001.

### Comprehensiveness

The mean Gwet’s AC1 score for interrater reliability for comprehensiveness was 0.89 (Supplementary File S1). The results of the comprehensiveness analysis are shown in Table 3 (by material type) and Table 4 (by material source). The average comprehensiveness of all materials was approximately 73.5% (SD = 14.7) and only 6.7% (*n* = 11) of materials included all required content. The materials included relatively more contents, the coverage of two topics was low: the importance of vaccination before first sexual intercourse and benefits of vaccination for men (24.4%, *n* = 40, 17.9%, *n* = 25, respectively).

A comparison of comprehensiveness by material type and material source is shown in Table 5. The comparison of material type showed that the comprehensiveness score of local government mailings was significantly higher than that of websites (median: 87.5, 66.7, respectively, adjusted *P* < 0.001). The comparison by material source showed that the score for academic materials was higher than that for government materials (median: 88.9, 75.0, respectively, adjusted *P* = 0.001) and higher than that for hospital materials (median: 88.9, 66.7, respectively, adjusted *P* = 0.014).

## DISCUSSION

The results of this study indicate that most Japanese HPV vaccination educational materials are difficult to understand and act upon and do not include all the necessary content about HPV vaccination. The mean understandability and actionability scores did not reach the 70% cutoff, a pattern similar to that of a previous study that assessed HPV vaccination counseling materials in the USA and that found understandability and actionability scores of 41.7% and 20.7%, respectively (Chhabra et al. 2018). These findings suggest that lay people considering HPV vaccination may have difficulty understanding and acting upon vaccination materials because of the lack of understandability and actionability of such materials.

Regarding understandability, the materials we analyzed mainly presented numbers as percentages rather than frequencies, did not include a summary, and used minimal (if any) visual aids. Regarding actionability, few materials indicated clear steps of action the user could follow by step, making it difficult for users to identify appropriate behaviors. As mentioned above, there has been a vaccination hiatus in Japan for several years. Online and offline materials are the only channels for informing the target population of the importance of HPV vaccination. Therefore, improving such materials with reference to the PEMAT-P is essential.

Regarding the comprehensiveness of materials, two types of content had low scores: the importance of vaccination before sexual debut and benefits of vaccination for men. These findings are consistent with those of a previous study (Chhabra et al. 2018). In recent years, the global incidence of HPV-related oropharyngeal cancer has increased, and the incidence of oropharyngeal squamous cell carcinoma in men is now greater than the incidence of cervical cancer in the UK (Lechner et al. 2019). Therefore, HPV vaccination is necessary for both women and men. In the materials evaluated in the present study, the importance of vaccination before sexual intercourse was often implied rather than expressed explicitly (e.g. expressed as ‘before getting infected with HPV’ not ‘before sexual debut’). As HPV infection generally occurs soon after sexual debut (Markowitz et al. 2014), it is important to provide explicit information about the importance of HPV vaccination before first sexual intercourse.

The comparison of materials by type showed that the local government mailings had the highest ratings for understandability, actionability, and comprehensiveness. These mailings included titles that were more obviously related to the topic, more direct expressions to urge the reader to take action, and more specific information to enhance the reader’s actions compared with the websites. However, the local government mailings sometimes used terms or expressions that were difficult to understand. This was because the mailings were based on the MHLW leaflets, which contained complex terminology and expressions. The comparison of materials by source showed that the government materials had the highest ratings for understandability and actionability, as found in a previous study (Cajita et al. 2017), but the academic materials had the most comprehensive content. There was a substantial difference in the use of technical terms between the government materials and academic materials. The latter tended to use medical terms without definition whereas the government materials used plain and general language not need definition. Academic materials provided a wide range of content, including a variety of statistical information, but this increased the size of the documents and necessitated the use of long sentences. Health service professionals and institutions should try to use simpler language to improve the understandability of health content.

This study had several limitations. First, we evaluated the understandability and actionability of vaccination materials using the PEMAT-P, and assessed the comprehensiveness of the content. However, we were unable to assess other important aspects of the content, such as readability, reliability, and persuasiveness. Second, it may be difficult to generalize the findings because of the following limitations of the materials assessed: we only examined Japanese materials, only mailings from local governments with populations of more than half a million were included, we did not include clinical materials used in medical institutions, and we did not assess all webpages that contained HPV vaccination educational materials. Finally, there was a possibility that the evaluations were subject to coding bias, although we did assess interrater reliabilities.

Despite these limitations, to our knowledge, this is the first study to evaluate the understandability, actionability, and comprehensiveness of Japanese HPV vaccination educational materials, and the findings have several implications for improving such materials. The following recommendations could help to improve the understandability, actionability, and comprehensiveness of HPV vaccination educational materials:

(1) Ensure that all medical terms are defined, if possible.
(2) Express numbers using frequencies or graphs, not percentages.
(3) Summarize the important main points at the end.
(4) Keep the text brief and simple, and use visual aids (e.g. illustrations and charts) that help the reader understand the content or take action.
(5) Provide a specific sequence of actions that readers could take.
(6) Present each type of content simply but include all necessary information, such as the importance of vaccination before sexual debut and the benefits of vaccination for men.
(7) To improve the actionability of website materials, use direct expressions, provide clear procedures, and include comprehensive content.
(8) To improve the understandability of academic materials, avoid very long documents and complex charts.

## CONCLUSION

Although it is essential that the eligible population understands the benefits and risks of HPV vaccination, we found that most Japanese HPV vaccination educational materials were insufficiently understandable and actionable. Few materials included sufficient information to enable decision-making about HPV vaccination. Most materials lacked definitions of medical terms, clear explanations of numbers, content summaries, explicit steps of action, and visual aids, and failed to mention the importance of vaccination before sexual debut and the necessity of vaccination for men. Regarding material type, the actionability and comprehensiveness of the websites were lower than that of local government mailings. Regarding material source, the understandability of hospital materials and academic materials was lower than that of government materials. There is a need to improve the actionability, understandability, and comprehensiveness of Japanese HPV vaccination educational materials.

## Supplementary Material

daaf034_suppl_Supplementary_Files_1

## Data Availability

The data underlying this article were provided by the local governments by permission. Data will be shared upon request to the corresponding author with the permission of the local governments.
