# Supplementary material for: Evaluating the understandability and actionability of Japanese human papillomavirus vaccination educational materials on cervical cancer
Source: Health Promot Int. 2025 Apr 23;40(2):daaf034. doi: 10.1093/heapro/daaf034 (PMC12015605; doi:10.1093/heapro/daaf034)
Supplement: daaf034_suppl_Supplementary_Files_1 [file daaf034_suppl_supplementary_files_1.docx]

**Supplementary file 1. The mean Gwet’s AC1 interrater reliability scores**

| Item | | | Gwet's AC1 | 95%CI |
| --- | --- | --- | --- | --- |
| **Understandability** | | | **0.81** | **(0.77, 0.85)** |
| Content | |  |  |  |
|  | 1 | The material makes its purpose completely evident | 0.68 | (0.41, 0.94) |
|  | 2 | The material does not include information or content that distracts from its purpose | 0.86 | (0.71, 1.00) |
| Word choice & style | | |  |  |
|  | 3 | The material uses common, everyday language | 0.77 | (0.54, 0.99) |
|  | 4 | Medical terms are used only to familiarize audience with the terms. When used, medical terms are defined | 0.66 | (0.39, 0.92) |
| Use of numbers | | |  |  |
|  | 5 | Numbers appearing in the material are clear and easy to understand | 0.86 | (0.73, 1.00) |
|  | 6 | The material does not expect the user to perform calculations | 1.00 | (NaN, NaN) |
| Organization | | |  |  |
|  | 7 | The material breaks or "chunks" information into short sections | 0.90 | (0.78, 1.00) |
|  | 8 | The material's sections have informative headers | 0.75 | (0.54, 0.96) |
|  | 9 | The material presents information in a logical sequence | 0.94 | (0.84, 1.00) |
|  | 10 | The material provides a summary | 0.97 | (0.90, 1.00) |
| Layout & design | | |  |  |
|  | 11 | The material uses visual cues (e.g., arrows, boxes, bullets, bold, larger font, highlighting) to draw attention to key points | 0.82 | (0.62, 1.00) |
| Use of visual aids | | |  |  |
|  | 14 | The material uses visual aids whenever they could make content more easily understood (e.g., illustration of healthy portion size) | 0.90 | (0.78, 1.00) |
|  | 15 | The material's visual aids reinforce rather than distract from the content | 0.67 | (0.45, 0.89) |
|  | 16 | The material's visual aids have clear titles or captions | 0.72 | (0.51, 0.93) |
|  | 17 | The material uses illustrations and photographs that are clear and uncluttered | 0.70 | (0.48, 0.91) |
|  | 18 | The material uses simple tables with short and clear row and column headings | 0.70 | (0.49, 0.91) |
| **Actionability** | | | **0.85** | **(0.80, 0.91)** |
|  | 19 | The material clearly identifies at least one action the user can take | 1.00 | (NaN, NaN) |
|  | 20 | The material addresses the user directly when describing actions | 0.78 | (0.47, 0.96) |
|  | 21 | The material breaks down any action into manageable, explicit steps | 0.78 | (0.56, 0.99) |
|  | 22 | The material provides a tangible tool (e.g., menu planners, checklists) whenever it could help the user take action | 0.60 | (0.32, 0.89) |
|  | 23 | The material provides simple instructions or examples of how to perform calculations | 1.00 | (NaN, NaN) |
|  | 24 | The material explains how to use the charts, graphs, tables, or diagrams to take actions | 0.84 | (0.69, 1.00) |
|  | 25 | The material uses visual aids whenever they could make it easier to act on the instructions | 0.93 | (0.83, 1.00) |
| **Comprehensiveness** | | | **0.89** | **(0.85, 0.93)** |
|  | 1 | Overview of cervical cancer | 0.97 | (0.91, 1.00) |
|  | 2 | HPV vaccine as cancer prevention | 1.00 | (NaN, NaN) |
|  | 3 | Emphasis of grade of 6–10 recommendation | 0.93 | (0.84, 1.00) |
|  | 4 | Importance of vaccination before first sexual intercourse | 0.76 | (0.56, 0.97) |
|  | 5 | Information of safety and efficacy | 0.82 | (0.64, 1.00) |
|  | 6 | Explanation of three times vaccination | 0.97 | (0.90, 1.00) |
|  | 7 | No charge for vaccinations | 0.78 | (0.56, 0.99) |
|  | 8 | Vaccination and benefit for men | 0.84 | (0.69, 1.00) |
|  | 9 | Side effects or adverse reactions | 0.87 | (0.72, 1.00) |
